# Protocol for a mixed-methods longitudinal study to identify factors influencing return to work in the over 50s participating in the UK Work Programme: Supporting Older People into Employment (SOPIE)

**DOI:** 10.1136/bmjopen-2015-010525

**Published:** 2015-12-16

**Authors:** Judith Brown, Joanne Neary, Srinivasa Vittal Katikireddi, Hilary Thomson, Ronald W McQuaid, Alastair H Leyland, John Frank, Luke Jeavons, Paul de Pellette, Sibel Kiran, Ewan B Macdonald

**Affiliations:** 1 Healthy Working Lives Group, Institute of Health and Wellbeing, College of Medical, Veterinary and Life Sciences, University of Glasgow, Glasgow, UK; 2 MRC/CSO Social and Public Health Sciences Unit, Institute of Health and Wellbeing, College of Medical, Veterinary and Life Sciences, University of Glasgow, Glasgow, UK; 3 Stirling Management School, University of Stirling, Stirling, UK; 4 Scottish Collaboration for Public Health Research and Policy (SCPHRP), University of Edinburgh, Edinburgh, UK; 5 Ingeus, Granite House, Glasgow, UK

**Keywords:** PUBLIC HEALTH, QUALITATIVE RESEARCH, STATISTICS & RESEARCH METHODS, OCCUPATIONAL & INDUSTRIAL MEDICINE, REHABILITATION MEDICINE

## Abstract

**Introduction:**

Increasing employment among older workers is a policy priority given the increase in life expectancy and the drop in labour force participation after the age of 50. Reasons for this drop are complex but include poor health, age discrimination, inadequate skills/qualifications and caring roles; however, limited evidence exists on how best to support this group back to work. The Work Programme is the UK Government's flagship policy to facilitate return to work (RTW) among those at risk of long-term unemployment. ‘Supporting Older People Into Employment’ (SOPIE) is a mixed-methods longitudinal study involving a collaboration between academics and a major Work Programme provider (Ingeus). The study will investigate the relationship between health, worklessness and the RTW process for the over 50s.

**Methods and analysis:**

There are three main study components. Embedded fieldwork will document the data routinely collected by Ingeus and the key interventions/activities delivered. The quantitative study investigates approximately 14 000 individuals (aged 16–64 years, with 20% aged over 50) who entered the Ingeus Work Programme (referred to as ‘clients’) in a 16-month period in Scotland and were followed up for 2 years. Employment outcomes (including progression towards work) and how they differ by client characteristics (including health), intervention components received and external factors will be investigated. The qualitative component will explore the experiences of clients and Ingeus staff, to better understand the interactions between health and (un)employment, Work Programme delivery, and how employment services can be better tailored to the needs of the over 50s.

**Ethics and dissemination:**

Ethical approval was received from the University of Glasgow College of Social Sciences Research Ethics Committee (application number 400140186).

**Results:**

Results will be disseminated through journal articles, national and international conferences. Findings will inform current and future welfare-to-work and job retention initiatives to extend healthy working lives.

**Strengths and limitations of this study**

- Our collaboration with Ingeus provides a unique opportunity to carry out high-quality innovative research, making use of a large rich data set to investigate a hard-to-reach population.
- Quantitative and qualitative components will track progress of individuals across their 2-year engagement with the Work Programme.
- The quantitative analysis will rely on routine operational data collected during the 2-year period of the intervention, but we will also explore the feasibility of linkage of Work Programme data to National Health Service (NHS) Scotland Information Services Division (ISD) health data.

## Introduction

Given the increase in life expectancy, and the growing percentage of individuals aged 65 and over, compared with the working-age population,^1^ older workers are an emerging priority group for policy makers.^2^ ^3^ In the UK, there are 7.9 million people aged 50–64 in employment and over one million workers over the age of 65 with 250 000 more people aged over 50 in work compared with a year ago. However, unemployment in this group is considerably higher, with a dramatic drop in labour force participation occurring after age 50. Employment rates decline sharply from over 80% of 50-year olds being in work, to around 60% of 60-year olds and just 30% of 65-year olds.^4^ Recent reports found around one million people over 50 have had to leave work against their will and would like to be working if appropriate opportunities were available.^5^ ^6^ Despite the recent growth in jobs, half of the people aged 45–70 who experienced unemployment during the past 5 years are not currently working.^7^

Work is an important determinant of health and health inequalities, with being out of work associated with poorer health outcomes and re-employment with improvements in health and well-being.^8–19^ However, debate continues over whether unemployment causes deterioration of health and/or whether those at higher risk of unemployment were in poorer health prior to becoming unemployed.^18^ ^20^ This is especially the case when looking at individuals over 50 years (defined as ‘older workers’), as there are difficulties in separating the impact of biological ageing from the impact of unemployment and from health selection effects (eg, ill health leading to early voluntary retirement).^18^

Previous research suggests increased employment in older workers may improve physical functioning and mental health.^21–23^ However, individuals of older working age are less likely to regain employment after job loss and are at increased risk of chronic health conditions which contribute to job loss and may make re-employment difficult.^24–27^ In addition, this age group may encounter other barriers including age discrimination from employers in recruiting and retaining staff,^28–30^ skills gaps (especially in IT),^29^ and caring responsibilities (eg, for grandchildren or other family members).^31^ Some individuals may experience multiple interacting or overlapping difficulties which may be difficult to resolve in isolation.^32^ ^33^

The Work Programme is the UK Government's flagship initiative to help those more detached from the labour market to enter employment and was launched throughout Great Britain in June 2011 as part of a sweeping programme of welfare reforms.^34^ As a result, more people are required to either seek work or to undertake some form of work-related activity as a condition of receiving benefit. Both those unemployed and those out of work due to health reasons are required to participate in the Work Programme, and others are able to volunteer to use the service at various stages of their claims process depending on their circumstances.^35^ Individuals engaging in the Work Programme are referred to in policy documents as ‘participants’, ‘clients’ or ‘customers’; this paper will use the term ‘clients’^i^. The 2-year Work Programme is delivered by a small number of large prime contractors at a regional level, who may subsequently subcontract regional and local delivery to a range of private, public and voluntary or community sector providers.^36^ Work Programme delivery in Scotland is delivered through two contracts held by Ingeus and Working Links.^35^ Contractors are paid on outcomes, defined by the length of the employment sustained by clients helped into work.

Referrals to the Work Programme are determined by the Department for Work and Pensions (DWP) and managed by Jobcentre Plus. DWP has a ‘black box’ approach to Work Programme delivery.^34^ This means DWP do not specify the type or level of support providers give to people seeking work, but instead allow providers scope to design provision with minimum service standards agreed with contractors. This only enables providers’ service delivery model flexibility until the contract is signed. After which, they are contractually bound to adhere to their model, unless changes are agreed with DWP. A rigorous study of policy effectiveness is challenging given the evolving nature of the client groups, the ongoing development of the components and delivery mechanisms of the Work Programme and the UK-wide implementation preventing identification of a reliable control group.^37–39^ The elements of support provided to Work Programme clients vary within the 2-year period of the service, but typical features include regular contact with an employment advisor, an assessment of the employment needs of the individual, help with searching for suitable jobs, preparing for interview, IT support and training through Employability Workshops.

A 2014 evaluation of the Work Programme, detailing client experiences found that after 2 years on the programme, 67% of people were not in work, and they were more likely to be male, older than 55, have health conditions, few qualifications and no recent work experience.^32^ There was also limited evidence of ‘creaming’ (prioritising those most likely to get employment) and ‘parking’ (of those with more substantial barriers to work, including many older workers).^40^ ^41^ A recent DWP evidence review on supporting the return to work (RTW) of the over 50s recognised health as a significant work barrier for older people, but also commented that little was known about their experiences of participating in RTW interventions.^42^ Related to this, they also suggested that programme monitoring should measure softer outcomes like ‘distance travelled’ and ‘movement towards’ the labour market since, while clients may not have obtained paid employment, they may have garnered skills in CV writing, interviews and self-confidence. The current study seeks to fill these identified gaps in the evidence base.^42^

### Aims

This mixed-methods longitudinal study addresses two broad research aims and a number of more specific research objectives (table 1). The first aim is to understand the different work and health trajectories experienced by clients during their engagement with the Ingeus Work Programme and how these differ by stage of the lifecourse and multiple dimensions of socioeconomic position. The second aim is to investigate the relationships between health, worklessness, the RTW process and the sustainability of employment in the older age working population (aged 50 and over and referred to as ‘over 50s’).

**Table 1:** Aims and RO

Aim	Related RO	Method utilised to answer RO
RA 1. To understand the different work and health trajectories experienced by clients during their engagement with the Ingeus Work Programme and how these differ by stage of the lifecourse and multiple dimensions of socioeconomic position	RO 1a. Map the detailed processes that clients pass through in the WP including developing an understanding of how advisers assess needs, determine the components of service delivery received and determine the processes by which advisers follow-up clients to assess their RTW and health outcomes	Embedded and qualitative components
	RO 1b. Describe the experiences, perceptions and behaviours of WP clients and staff and the influence of these experiences on clients’ sustainable RTW and health (with a particular focus on the over 50s)	Qualitative component
	RO 1c. Analyse Ingeus’ routinely collected data, and qualitative data from clients and service providers, to investigate factors which promote movement towards work, maintain sustainable RTW and improve health and well-being, with a particular focus on the over 50s	Qualitative and quantitative components
RA 2. To investigate the relationships between health, worklessness, the RTW process and the sustainability of employment in the older age working population (over 50 years)	RO 2a. Identify details of data routinely collected by Ingeus during the client journey, (including assessing its quality and completeness for research purposes)	Embedded component
	RO 2b. Match client data (using datazones) to secondary data (eg, neighbourhood statistics, SIMD) so as to investigate influences of labour market conditions, travel to work opportunities in the quantitative analysis	Quantitative component
	RO 2c. Explore the feasibility of linkage of Ingeus data to ISD health data (eg, death records, acute hospital discharges, psychiatric hospital admissions, cancer registrations and prescriptions data)	Quantitative component

ISD, Information Services Division; RA, research aim; RO, research objective; RTW, return to work; WP, Work Programme.

This study has been funded as part of the Medical Research Council (MRC) Lifelong Health and Wellbeing Extending Working Lives Partnership Awards. The main remit of this funding stream is to support cross-sector collaborations between academics and public/private employers or stakeholder organisations to address research challenges and employers’ needs associated with promoting health and well-being in the older workforce.^43^ This research will be undertaken by a partnership between Ingeus, a welfare-to-work provider, the academic team and the data controller, DWP.^44^ ^45^ This study will make use of Ingeus routinely collected individual-level data to describe the characteristics of those who RTW by age and health status and establish the extent that differences in health and social factors mediate the relationship between age and RTW. No alternative routine data are available on this population group in the UK as the sample sizes within existing social survey data (even the large Understanding Society survey)^46^ are small in relation to the long-term workless, particularly given high rates of non-response among this group. Secondary data will be matched to individuals to allow consideration of external factors, such as local labour market conditions. Additionally, this study will interview Work Programme clients to explore their experiences prior to, and during the Work Programme. This project will detail the lived experiences of a hard-to-reach population currently engaged in the Work Programme, and will highlight the interactions between health, employment history and the RTW process. This study aspires to produce specific recommendations for ensuring this group is better served in future.

## Methods

### Study design

This is a mixed-methods longitudinal study making innovative use of data routinely collected by a deliverer of a welfare to Work Programme (matched to administrative data on labour market conditions and local Work Programme office areas), combined with in-depth interviews of providers and longitudinal qualitative interviews with clients. There are three main components to the project—embedded fieldwork, quantitative study and qualitative study. Preliminary findings from the quantitative analysis will inform later phases of qualitative data collection and hypotheses derived from qualitative analysis will be tested in the quantitative component.

#### Embedded fieldwork

Although the research team sought to gain an initial understanding of the Ingeus data collection procedures prior to the project, the first stage of the research will be to fully document the data Ingeus collect, the key interventions/activities and the content of other core services offered (RO 1a and 2a in table 1). The researchers will be embedded in the Ingeus offices in Scotland (Livingston, Glasgow, Edinburgh and Musselburgh), observing client appointments for the first 6 months of the project. Detailed fieldwork notes on Ingeus client assessment and adviser decision-making processes will be made.

#### Quantitative study

##### Study population

**Figure 1:**
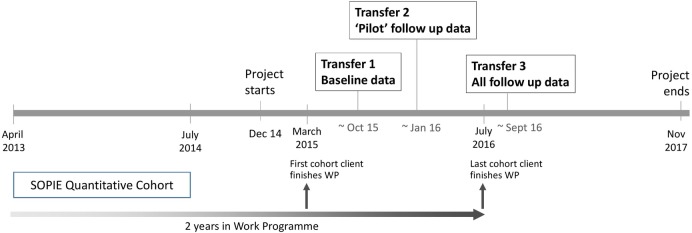
Timing of SOPIE quantitative cohort and data transfers (SOPIE, Supporting Older People Into Employment; WP, Work Programme.

The quantitative study population will be 14 265 Ingeus clients who entered the Work Programme in Scotland between the 1 April 2013 and the 31 July 2014. Of these clients, 2846 are aged over 50 (20% of total). This cohort will be followed up longitudinally for the 2 years they are engaged in the Ingeus Work Programme (figure 1).

##### Study variables

###### Baseline variables

Clients are assessed by an Ingeus employment advisor and complete a baseline assessment. This assessment helps the adviser plan and deliver interventions suitable for each client to assist in their RTW, based primarily on their assessed proximity to employment. Baseline variables collected are shown in table 2.

**Table 2:** Variables to be used in quantitative component

Data collected	Examples	Further information
Baseline measures	Age, sex, ethnicity, benefit and employment history, reason for leaving last job, health condition (s), health concerns that affect ability to work, job goal, motivation to find work, education level, housing status, parental status, carer status, data zone	Collected by employment advisor following referral to Work Programme Datazone computed by Ingeus from client's postcode
Intervention components	*Employability Workshops* (eg, ‘interview practice’, ‘letter writing support’, ‘online job search support’) *Employer Services* (eg, ‘job finding’, ‘prescreening’, ‘sectoral routeway training’ eg, food hygiene and Construction Skills Certification Scheme tests) *Health and Wellbeing Service (HWS) workshops* (eg, health-specific, lifestyle and well-being, weekly exercise, work-specific and health education workshops) Health specific questionnaires PHQ9 (9-item depression-specific questionnaire) PHQ15 (15-item Somatic Symptom Severity scale) GAD7 (7-item Generalized Anxiety Disorder scale) Confidential Client Questionnaire (CCQ, generic covers physical and mental health and work-related health)	Attendance at the workshop/appointment, the date and where appropriate if the intervention was completed will be provided Questionnaires only collected for those clients who engage with HWS (pre and post measures may be available)
Distance travelled and progression towards work	Ingeus use a CMF, to track the progress of clients through the Work Programme towards work. There are eight recordable ordinal scale stages to the CMF and this will be used to indicate a client's ‘distance travelled’ and movement towards the labour market	Running count of the times that a client has been moved from one CMF category to another. Will also be provided with the first and latest CMF rating including dates
Milestones and Job outcomes	Job start, 13-week job outcome, 26-week job outcome Sustainment outcome, job title, type of employment started, type of contract, name of employer, location of job	Variables available if client enters work and sustains in work
Area characteristics	Local level of multiple deprivation (which may influence factors such as peer pressure, role models) as well as potential employer discrimination Transport links and distance to key sources of information and training, for example, distance to further education; unemployment levels Local demand measures including employment centres (part time, low paid workers are less likely to travel far, so travel-to-work data are insufficient) and vacancy data (although local Job Centre Plus vacancy data need to be treated with caution) distance to health services Various forms of urban area, rurality and remoteness measures	Completed by research team using other data for example, SIMD, The Scottish Government Urban Rural Classification (eightfold)

CMF, caseload management framework; HWS, Health and Wellbeing Service; SIMD, Scottish Index of Multiple Deprivation.

###### Intervention components

The intervention components include Employer Services, Employability Workshops and Health and Wellbeing Services (HWS) (table 2). The voluntary HWS provides advice on how to understand, cope with and manage existing health-related conditions or issues without ‘medicalising’ the conditions.^47^ The service is not therapy, treatment or counselling, but it is delivered by clinically qualified practitioners (physiotherapists, occupational therapists and psychologists). Issues they address typically include loss of self-confidence, low mood, anxiety related to returning to work, loss of structure and routine. Initial triaging is performed by the health advisors, and clients are then directed to workshops which best fit their requirements. The workshops include relaxation, physical exercise classes and confidence building (table 2).

###### Job outcome and distance travelled variables

Ingeus also use a ‘caseload management framework’ to track the progress of clients through the Work Programme, and this will be used to indicate a client's ‘distance travelled’ and movement towards the labour market. Ingeus are paid almost entirely by results (job outcome fee and sustainment fees), and thus there are robust data on job outcomes during the Work Programme and for 2 years post-RTW (details in table 2).

###### Matching to area characteristics

Analyses of area characteristics will be conducted using data zones which are the key small-area statistical geography in Scotland.^48^ Data zones are groups of 2001 Census output areas and have populations of between 500 and 1000 household residents. Ingeus will determine the data zone from the client's postcode. Where there are less than three clients in a data zone, the data for those clients will be combined into an adjacent data zone with more clients, so as to maintain anonymity. The research team will add geocoded information relating to area of residence at different spatial scales including the different domains of the Scottish Index of Multiple Deprivation (SIMD)^49^ at the level of data zone, local demand for relevant types of job and job density at the level of local authority (LA; table 2).

##### Data transfers

While Ingeus are responsible for data collection and Work Programme delivery, the data controller is the government department responsible for commissioning the programme, the DWP. DWP have agreed three transfers of anonymised Supporting Older People Into Employment (SOPIE) cohort data (figure 1). The first transfer will be the baseline assessment data (Autumn 2015), the second transfer will be ‘pilot’ follow-up data on a sample of the completed SOPIE cohort (early 2016) and the third transfer will be the follow-up data on all the SOPIE cohort (early Autumn 2016).

##### Sample size

A useful rule of thumb for survival analysis power calculations, covering multivariate Cox and parametric regression methods, is that the data set should include 10–20 participants with uncensored outcomes (RTW in this study) per independent variable ever modelled.^50^ Data provided by Ingeus estimates that 496 job seeker allowance (JSA) clients aged over 50 and 160 employment support allowance (ESA) clients aged over 50 will have a least one job start in their time in the Work Programme (Ingeus. Personal Communication (on 1st October 2015), 2015). With 496 such participants expected in the JSA group, there will be ample power for modelling the influence on RTW of at least 25, and perhaps up to 50 independent variables, which more than covers those expected to have substantial policy or practice interest (ie, 0.9> hazard rate ratios or 1.1< hazard rate ratios, a commonly used benchmark), based on previous studies.^51^ ^52^ The ESA group, with only 160 participants providing power, may not be large enough to reliably model the effects of more than 8–16 such variables within that ESA category. However, by merging the two categories and using a dummy variable for JSA versus ESA status, this problem should be largely overcome.

##### Data linkage

In order to investigate potential health impacts, including longer term benefits, of engaging with the Work Programme, we will explore the feasibility with DWP and Ingeus of carrying out linkage of Work Programme data to National Health Service (NHS) Scotland Information Services Division (ISD) health data. We would use the Electronic Data Research and Innovation Service (eDRIS) and the Administrative Data Research Network.

#### Qualitative study

The qualitative study aims to explore the interaction between age, health and employability across the lifecourse and investigate how health contributes to, and is impacted by, worklessness. Qualitative fieldwork will not cover the entirety of Scotland. Seven study areas have been selected, with the agreement of Ingeus, to ensure coverage of a diverse range of Scottish areas (including urban, suburban and rural communities).

Given time constraints and the 2-year follow-up of the quantitative cohort, it is not possible for the qualitative study to sample from the same cohort as the quantitative study. Participants in the qualitative study will have entered the Work Programme between June 2014 and June 2015. This reflects the longitudinal nature of the qualitative study, as one of the aims of this study is to follow clients as they progress through the Work Programme. The first wave of the qualitative study will consist of longitudinal in-depth semistructured interviews with 40 purposively selected participants aged over 50 years who are in their first year of the Work Programme. The study will seek to reinterview them after approximately 12 months. By conducting two waves of data collection, 1 year apart, the study is designed to provide insights into participants’ experiences of the entire 2-year Work Programme, as well as capture changing understandings, perceptions and expectations.

The study will also seek to interview 15 Work Programme practitioners, to better understand service delivery, what they believe are the main barriers and facilitators faced by the over 50s in returning to work, and their experiences working as front-line staff in the Work Programme. This will enable differing views on the same phenomena to be compared and contrasted.^53^

##### Qualitative participant recruitment

Our recruitment methods were negotiated and formally agreed with DWP, Ingeus and University of Glasgow. Recruitment of clients and practitioners differs and is detailed below.

###### Work Programme client recruitment

**Figure 2:**
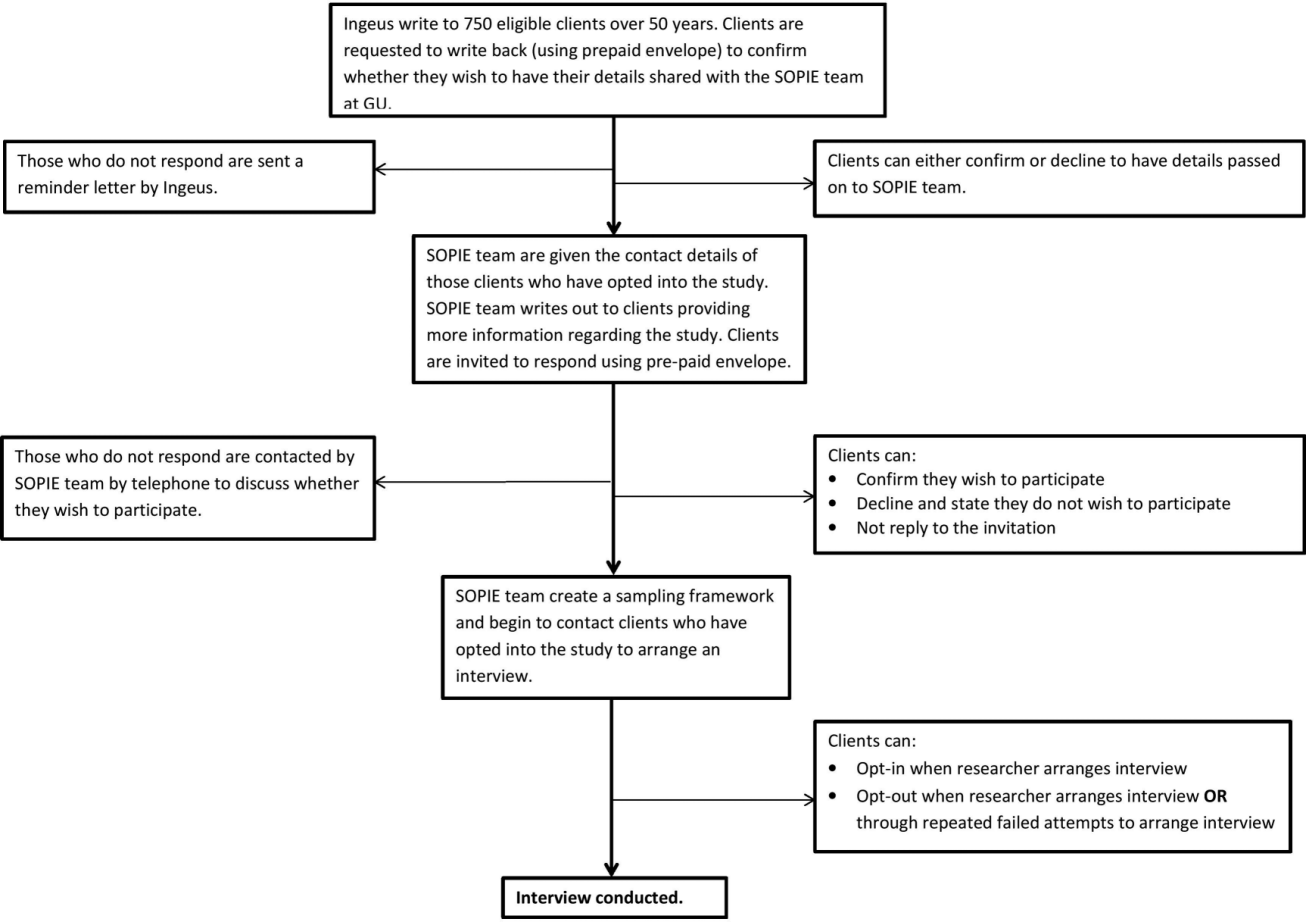
Flow diagram of recruitment strategy (SOPIE, Supporting Older People Into Employment).

Under this agreement, consent of participants will be obtained through two stages: consent to share contact details with University of Glasgow, and consent to participate in the study (figure 2). The first stage will be conducted by Ingeus, on behalf of DWP, and will involve writing to eligible clients to ask permission to share their contact details with University of Glasgow. Once initial consent has been gained, the University of Glasgow research team will contact the clients to provide further information regarding the study. As detailed below, not all individuals who opt-in will be interviewed, with inclusion based on the sampling framework below.

###### Work Programme practitioner recruitment

All Ingeus team managers throughout Scotland will be emailed a recruitment poster and to ask for them to pass on details of the study to their employees. To ensure anonymity and confidentiality, interested staff will be asked to email JN directly, and not include their Ingeus team manager.

##### Sampling

###### Work Programme client interviews

Participants will be sampled to ensure diversity of key demographic, employment and Work Programme characteristics. While the quantitative and qualitative cohorts cover different time periods, it is anticipated that the qualitative sampling framework will reflect the wider demographic trends in the quantitative data. Therefore, the sampling framework will use similar ratios of gender, age ranges and location (eg, urban, suburban, rural). The sampling framework will also take account of the length of time on the programme.

As stated above, the qualitative study will sample from a cohort of clients over 50 years who joined the Work Programme between June 2014 and 2015; therefore, ‘length of time on the Work Programme’ will cover the first 15 months of Work Programme engagement. We will also sample those who are currently engaged with the Work Programme but are currently in work, providing their contact details have not changed. Recruitment will continue until adequate diversity in the sample has been achieved and no new themes are being identified in the data.^54^ It is anticipated that approximately 40 participants will be interviewed at baseline, and at least 20 (accounting for attrition) at follow-up.

###### Work Programme practitioner interviews

Fifteen Work Programme practitioners will be interviewed from various locations throughout Scotland. We are aiming to speak to a wide range of practitioners currently involved in delivering the Work Programme, including front-line staff (those dealing with employment or health issues of clients), as well as those in more managerial roles (including office managers, and those facilitating positive links between the Work Programme and local business).

##### Interview process

###### Work Programme client interviews

Interviews will be conducted face-to-face in either the participant's home or a location that is suitable and accessible for them (eg, a library, university office). Interviews will take approximately 1–1½ h to complete. A semistructured topic guide will be used to ensure coverage of key topics, but also enable the fieldwork to be flexible, enabling a more informal atmosphere which may allow individuals to spontaneously tell and develop stories. The interview will focus on the interviewee's experience of: the Work Programme; the interaction between age, health, and work experience and employability across the lifecourse; and previous/current barriers and facilitators of employment and RTW. The topic guide will be reviewed by the qualitative study team after the first few interviews and intermittently thereafter, to ensure its appropriateness.

After a period of 12 months, repeat follow-up interviews will be conducted. It is anticipated that this follow-up interview will enable a richer understanding and exploration of trajectories in participants’ health and employment; changes in other socioeconomic determinants of health; and also document their continued engagement with the Work Programme, or their initial experiences post-Work Programme. The semistructured topic guide will cover some similar areas to the baseline interviews, but be adapted to allow more detailed exploration of themes that have emerged from the initial analysis.

###### Work Programme practitioner interviews

Interviews will be conducted face-to-face predominantly at University offices, although if this is not accessible for staff members, other locations will be sought. It was decided that interviews should not take place in the Work Programme offices as this would encroach on the guarantees of confidentiality and anonymity within the research. The interviews will explore work culture, the practitioner's day-to-day role and their understanding and perceptions of the barriers and facilitators experienced by the over 50s in returning to work.

##### Transcription

All interviews will be digitally recorded (with consent obtained by participants) and transcribed verbatim. Transcripts will be checked against the audio files for accuracy, and any references that may identify the participants or third parties will be removed.

The anonymisation process will differ for clients and practitioners. While clients will be allocated an agreed pseudonym, practitioners will be referred to by a generic job title. This will be agreed with all participants. Names of third parties and addresses will also be removed. Transcripts will be stored on a protected secure network, and will be coded using NVivo V.10 (qualitative data management software).

## Data analysis and dissemination

### Quantitative study

The relative frequencies of various patterns of RTW (or not) of the SOPIE cohort will be quantified, and the various intervention components provided, cross-classified by client characteristics relevant to programme success. Second, associations between individual and area characteristics, as well as intervention components, with sustainable RTW will be explored. Effect modification by age will be specifically sought.

The external environment in which the person lives, searches for jobs and receives health support will be matched to their individual characteristics (eg, education, heath) and personal circumstances (eg, housing types). Various forms of local labour demand and supply-side data will be investigated (as used by the researchers in previous studies).^55–57^

Analyses will focus on the association between individual characteristics and personal circumstances (such as age, sex, social class, employment history, pre-existing mental and physical health status, disability, engagement with Work Programme, qualifications, debt, caring responsibilities) and area characteristics and local labour market conditions (such as unemployment measures and job densities), and the outcomes of RTW and sustained RTW and any progression on the journey to work. Multilevel regression modelling will be used to take account of the clustering of individuals within data zones and LAs (travel to work areas are generally too large for travel patterns of low income and older workers, so LAs will be used) and to investigate the influence of area characteristics on RTW. Successive models will quantify the impact of sociodemographic characteristics, individual social factors, health and area characteristics on the outcomes, and we will report the extent to which these variables explain apparent differences between areas.

Given SOPIE's focus on extending working lives, the analysis will focus on the role of age. More specifically, we will compare those aged over 50 with an approximately equal number of those aged 40–49 to identify whether there is an age effect and whether the effects of any individual or area characteristics show differential age effects. This will enable us to determine whether the experiences of the older population differ from those that they are closest to in terms of age. We will base this on a series of multilevel regression models in which we will use fractional polynomials to model age as a continuous variable with a flexible functional form. We will then test the interaction between age and each of the relevant characteristics. Analyses will use logistic regression and, where appropriate, Cox regression models for time to event (RTW or length in work following return).

Random intercept models will be used to analyse the distance travelled along similar lines to the analysis described above for RTW. This will provide us with the individual and area characteristics associated with improvements in distance travelled and, in particular, will enable us to investigate the role of age.

### Qualitative study

Initially individual narratives will be explored by considering how each participant describes and explains how their experiences of health and employment interacted at various points in their working life. This initial analysis will also examine how the wider contexts of the participants lives (eg, family and social connections, and the wider macro-level context) may influence these transitions. This analysis will utilise narrative analysis techniques.^58^ ^59^ Narrative analysis focuses on the ways in which individuals explain and present their accounts of themselves and therefore construct, identity and use their stories to interpret the world.^60^ A coding diary will be used to highlight emerging themes that may cut across the different cases.

Following this, the analysis will identify patterns across the interviews. The second stage of analysis will use framework analysis,^61^ following three stages:

1. Using the coding diary from the narrative coding, a working analytical framework will be developed. This may require several iterations to account for all data.^62^
2. The analytical framework will facilitate development of a summary matrix which will summarise data by category or theme. A second researcher (HT or SVK) will independently analyse a subsection of the data to ensure a robust and transparent coding strategy is created.^63^
3. The matrix will aid identification of similarities and differences between the participants, and to identify cross-cutting themes across the sample.^62^

Double coding will be conducted for 10% of interviews to develop the coding framework.^64^ Insights emerging from the qualitative analysis will add value to the quantitative analysis, by pointing to fruitful areas of exploration within the larger quantitative data set. Similarly, potential explanations for findings from the quantitative analyses will be sought in the qualitative study component.

### Ethical considerations

There are three main categories of ethical consideration that need to be taken into account: safety of participant, safety of researcher and ethical independence of the study.

In terms of safety of the participant, attempts will be made to ensure no explicitly sensitive questions will be asked. However, as many of the client participants will be from vulnerable populations, having experienced long-term unemployment, long-term health issues, low income and other deprivations, there may be unprompted sensitive disclosures by participants. The qualitative researcher supported by the research team will draw on their previous experience working with vulnerable groups to ensure any admissions are dealt with in a sensitive manner. Anonymity and informed consent will be ensured.

In terms of researcher safety, a risk assessment has been carried out regarding fieldwork, and procedures have been put in place to minimise risk. These include the qualitative researcher carrying a security phone and panic alarm whenever she is conducting interviews. Debriefing sessions will be timetabled into the research period, and the researcher will have access to HWS at the University of Glasgow.

The third consideration concerns the study as a whole. While our study involves a partnership between the University of Glasgow and Ingeus, our researchers will ensure independence of the study is upheld and ethical standards applied. As noted in this protocol, Ingeus will share data with our research team, but they will have no influence with regard to analysis or publishing findings. The University of Glasgow is committed to the highest standards of academic quality and to ensure our work stands up to rigorous peer review.

### Dissemination

Communications will be achieved through: the creation of two active stakeholder groups, one in Scotland and one in London; social media (including website); Ingeus to the network of Work Programme providers and others involved in employment and in vocational rehabilitation; discussions with policymakers; and exploiting links with journalists in various media. The research partnership with Ingeus is particularly advantageous in view of Ingeus’ well-established processes for disseminating research and influencing policymakers.

Findings will be disseminated through peer-reviewed publications, national and international conferences and, at the final stage of the project, there will be a symposium for UK policy, academic and Work Programme leads to share findings, best practice and lessons learnt.

It is anticipated that the research will provide new information on the interventions, support and individual factors which assist the over 50s RTW and sustain work. The academic research will not only help inform how Ingeus can improve its interventions, but will also provide new information on how to prevent people having to leave work and extend working lives. This research will inform current and future welfare-to-work and job retention initiatives and improve their effectiveness in helping working age people extend their healthy working lives.

## Footnotes

i This decision was guided by Ingeus’ use of the same term.
